# Measurement with indefinite causal order and the Sagnac interferometer

**DOI:** 10.1098/rsta.2024.0447

**Published:** 2024-12-24

**Authors:** S. M. Barnett, S. Croke, S. Franke-Arnold

**Affiliations:** ^1^ School of Physics and Astronomy, University of Glasgow, Glasgow G12 8QQ, UK

**Keywords:** Sagnac interferometer

## Abstract

It has been shown that measurements involving indefinite causal order can be superior to those in which a sequence of operations occurs in a specified order. In optics, such measurements are realized naturally in a Sagnac interferometer. We show that such an arrangement can measure the solid angle (on the Poincaré sphere) enclosed by a sequence of unitary transformations of the polarization. This is the Pancharatnam–Berry phase. Extension from the classical or single-photon treatment to a fully quantized treatment allows the analysis of the interferometer for arbitrary quantum states of light.

This article is part of the theme issue ‘The quantum theory of light’.

## 1. Introduction

In the Schrödinger picture, an initial state evolves through the action of a unitary transformation. Thus an initial state, |ψ⟩, will become at a later time the state $\hat{U}|\psi \rangle$, where $\hat{U}$ is a unitary operator. If this initial dynamics is modified at a later time then the state will further evolve by the action of a second unitary operator to become $\hat{V}\hat{U}|\psi \rangle$. As is common in quantum theory, the operators $\hat{U}$ and $\hat{V}$ will not normally commute so that $\hat{V}\hat{U}$ is not the same as $\hat{U}\hat{V}$. This somewhat abstract picture may be formalized in the gate picture of quantum information processing, in which the two transformations, $\hat{U}$ and $\hat{V}$, are applied sequentially but in a specified order [1,2]. Indefinite causal order exploits the superposition principle to apply the transformations in a *superposition of orders* so that, for example, an initial state, |ψ⟩, might be transformed into [3,4]


(1.1) $$\begin{array}{lcr} & |\psi \rangle \to \frac{1}{\sqrt{2}}\left(\hat{U}\hat{V}+\hat{V}\hat{U}\right)|\psi \rangle . & \end{array}$$

It has been shown that this generalization of the usual causal gate structure can have advantages for several quantum communication and information processing tasks [5–9]. Some of these ideas have been demonstrated in experiments [10–19].

In optics, the Sagnac interferometer [20], with its two counter-propagating paths, forms a natural candidate to realize operations with indefinite causal order.^1^ If the two operations corresponding to the transformations enacted by $\hat{U}$ and $\hat{V}$ are placed inside the interferometer then the interfering counter-propagating fields will naturally experience the transformations in opposite orders. We exploit this possibility here to propose a demonstration of an indefinite-causal-order enabled measurement associated with two, in principle unknown, polarization transformations.

Our initial analysis treats the inferences that can be drawn from a set of single-photon interference experiments (or, equivalently, from an experiment using laser light [22]). It has long been appreciated, however, that the limits to interference measurements are imposed by the quantum theory of light [23,24]. As a prelude to exploring the effects of quantum noise in indefinite-causal-order experiments, we present a full quantum analysis of our proposed Sagnac device. This is based on Loudon’s analyses of the quantum theory of interferometers [25,26] and, in particular, the observation that a Sagnac interferometer can be thought of as a ‘folded’ Mach–Zender interferometer [27].

## 2. Metrology using indefinite causal order

We shall be concerned, here, with the potential benefits of using indefinite causal order in metrology. To this end, we discuss two techniques for measuring an area associated with sequences of unitary transformations. The first of these is the idea introduced by Zhao *et al*. in which an area in phase space associated with position and momentum displacements is measured [28]. The second is designed to measure an area on the Bloch or Poincaré sphere associated with a closed path generated by a pair of unitary transformations and their conjugates. It must be admitted that both measurements are somewhat contrived, but they are introduced only to illustrate the potential benefits of measurement with indefinite causal order.

### (a) Area in phase space

**Figure 1.**
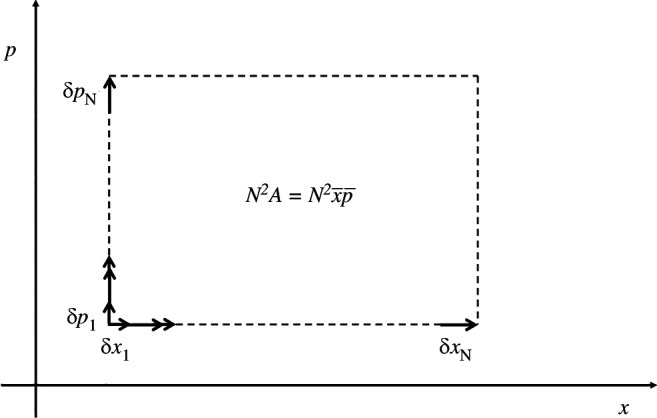
Phase space area A formed from the combination of N displacements in position and N displacements in momentum. The goal of the metrology task discussed in the text is to measure this area.


The key idea here is to use measurements exploiting indefinite causal order to measure a phase space area in the form of a product of average position displacement, $\bar{x}$, and momentum displacement, $\bar{p}$, so that $A=\bar{x}\bar{p}$ [28], shown in figure 1. These are formed in each case by the combination of N displacements:


(2.1) $$\begin{array}{lrlr} & \bar{x} & =\frac{1}{N}\sum_{j}x_{j}, & \end{array}$$

(2.2) $$\begin{array}{rl} \bar{p} & =\frac{1}{N}\sum_{k}p_{k} \end{array}.$$

The individual displacements are associated with a pair of sets of unitary operators of the forms:


(2.3) $$\begin{array}{lrlr} & {\hat{D}}_{x_{j}} & =e^{ix_{j}\hat{P}}, & \end{array}$$

(2.4) $$\begin{array}{lrlr} & {\hat{D}}_{p_{k}} & =e^{-ip_{k}\hat{X}}, & \end{array}$$

where $\hat{X}$ and $\hat{P}$ are the position and momentum operators for a quantum test particle, and we work with units in which ℏ=1. The effect of each is to shift, by a discrete interval, either the position or the momentum so that


(2.5) $$\begin{array}{lrlr} & {\hat{D}}_{x_{j}}\psi (x) & =\psi (x+x_{j}), & \end{array}$$

(2.6) $$\begin{array}{lrlr} & {\hat{D}}_{p_{k}}\tilde{\phi }(p) & =\tilde{\phi }(p+p_{k}), & \end{array}$$

where ψ(x) and $\tilde{\phi }(p)$ are, respectively, the position and momentum representation wavefunctions for our particle.

It was shown by Zhao *et al.* that a metrology protocol with definite causal order can achieve a precision scaling like $N^{-1}$ at best. A protocol exploiting indefinite causal order, however, gives a precision that scales as $N^{-2}$.

**Figure 2.**
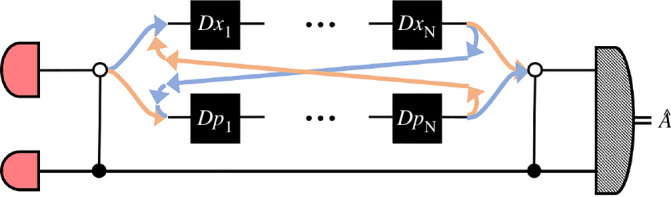
Schematic of indefinite causal order metrology scheme proposed by Zhao *et al*. (figure reproduced from [28]).

The proposed protocol proceeds as follows: a control qubit, initially in an equal superposition $\frac{1}{\sqrt{2}}\left(|0\rangle +|1\rangle \right)$ controls in which order the X and P displacements are applied. In one case, conditioned on the control qubit being in state |0⟩ all the X displacements are performed first, followed by all the P displacements. In the other case, when the control qubit is in state |1⟩, these are applied the other way round; all the P displacements and then all the X. A schematic is shown in figure 2 (figure taken from [28]). Thus, assuming an initial probe state |ψ⟩, the state of the joint control–probe system becomes


(2.7) $$\begin{array}{lrlr} & & \frac{1}{\sqrt{2}}\left(|0\rangle \prod_{k=1}^{N}{\hat{D}}_{p_{k}}\prod_{j=1}^{N}{\hat{D}}_{x_{j}}|\psi \rangle +|1\rangle \prod_{j=1}^{N}{\hat{D}}_{x_{j}}\prod_{k=1}^{N}{\hat{D}}_{p_{k}}|\psi \rangle \right) & \\ & & =\frac{1}{\sqrt{2}}\left(|0\rangle {\hat{D}}_{N\bar{p}}{\hat{D}}_{N\bar{x}}|\psi \rangle +|1\rangle {\hat{D}}_{N\bar{x}}{\hat{D}}_{N\bar{p}}|\psi \rangle \right), & \end{array}$$

where |ψ⟩ is the initial state of our test particle.

It follows from the canonical commutator $[\hat{X},\hat{P}]=i$ that [29,30]


(2.8) $$\begin{array}{lcr} & {\hat{D}}_{N\bar{x}}{\hat{D}}_{N\bar{p}}={\hat{D}}_{N\bar{p}}{\hat{D}}_{N\bar{x}}e^{-iN^{2}\bar{x}\bar{p}}, & \end{array}$$

and it follows that we can write our evolved state in the form:


(2.9) $$\begin{array}{lcr} & \frac{1}{\sqrt{2}}\left(|0\rangle {\hat{D}}_{N\bar{p}}{\hat{D}}_{N\bar{x}}|\psi \rangle +|1\rangle {\hat{D}}_{N\bar{x}}{\hat{D}}_{N\bar{p}}|\psi \rangle \right)=\frac{1}{\sqrt{2}}\left(|0\rangle +e^{-iN^{2}A}|1\rangle \right){\hat{D}}_{N\bar{p}}{\hat{D}}_{N\bar{x}}|\psi \rangle . & \end{array}$$

Thus, if the probe is measured in the $|\pm \rangle =\frac{1}{\sqrt{2}}\left(|0\rangle \pm |1\rangle \right)$ basis, the probability for obtaining each of the possible outcomes is


(2.10) $$\begin{array}{lcr} & P(\pm |A)=\frac{1}{2}\left(1\pm \cos(N^{2}A)\right). & \end{array}$$

This exhibits rapid oscillations as a function of the desired area $A=\bar{x}\bar{p}$, which is an indication of extreme sensitivity (for large N) and with it the potential for high precision.

There is an important piece missing from the above analysis, and this is the question of how to distinguish between A and $A\pm \frac{2n\pi }{N^{2}}$. In the experimental demonstration reported in [31], it is assumed that A is small so that $N^{2}A$ is known to be less than 2π. Without this promise, phase estimation techniques such as those described in [32] can be used to obtain an estimate.

We note that in [28] the displacements are considered to be a discrete number (N) of small displacements, but we can equivalently consider a total length l and displacement per unit length $\delta_{x}$ to give $x=l\delta_{x}$. In this case, given additional information from multiple measurements, the precision with which we can measure the product $\delta_{x}\delta_{p}$ scales as $l^{2}$ when indefinite causal order is used, as opposed to l for an experimental set-up in which the displacements are applied in a specified and definite order.

### (b) Area on the Bloch sphere

The metrology scheme in [28] measures an area in phase space. An equivalent in the qubit case would be measuring the area of a closed path on the Bloch sphere, i.e. a Pancharatnam–Berry phase [33–35]. Let us consider the path traced by the two possible orderings of two unitary operations $\hat{U}$, $\hat{V}$. Let us suppose that we can choose an initial state |ψ⟩ corresponding to a starting point on the sphere such that $\hat{U}\hat{V}$ and $\hat{V}\hat{U}$ result in the same endpoint, differing only by a global phase Ω/2, where Ω is the solid angle corresponding to the enclosed path. This means that the operations ${\hat{U}}^{†}{\hat{V}}^{†}\hat{U}\hat{V}$ acting on the state |ψ⟩ form a closed loop, and the global phase accumulated by traversing this loop is the Berry phase. Geometrically, it is clear that the antipodal point $|\psi^{⟂}\rangle$ accumulates an equal and opposite phase when acted upon by the same sequence of operations. Thus:


(2.11) $$\begin{array}{rl} {\hat{U}}^{†}{\hat{V}}^{†}\hat{U}\hat{V}|\psi \rangle & =e^{i\frac{\Omega }{2}}|\psi \rangle \\ {\hat{U}}^{†}{\hat{V}}^{†}\hat{U}\hat{V}|\psi^{⊥}\rangle & =e^{-i\frac{\Omega }{2}}|\psi^{⊥}\rangle . \end{array}$$

It is interesting to note that for an arbitrary state $|\phi \rangle =\alpha |\psi \rangle +\beta |\psi^{⟂}\rangle$, the unitary operations produce:


(2.12) $$\begin{array}{lcr} & \langle \phi |{\hat{U}}^{†}{\hat{V}}^{†}\hat{U}\hat{V}|\phi \rangle =|\alpha {|}^{2}e^{i\frac{\Omega }{2}}+|\beta {|}^{2}e^{-i\frac{\Omega }{2}}=\cos\;\frac{\Omega }{2}+i\;\sin\;\frac{\Omega }{2}(|\alpha {|}^{2}-|\beta {|}^{2}). & \end{array}$$

In other words, the real part of the expectation value of ${\hat{V}}^{†}{\hat{U}}^{†}\hat{V}\hat{U}$ in *any state* |ϕ⟩ is independent of the state, and is given by $\cos\;\frac{\Omega }{2}$. This suggests that it might be possible to measure the Berry phase without knowing the state |ψ⟩ and, indeed, without knowing the two unitary operators $\hat{U}$ and $\hat{V}$.

**Figure 3.**
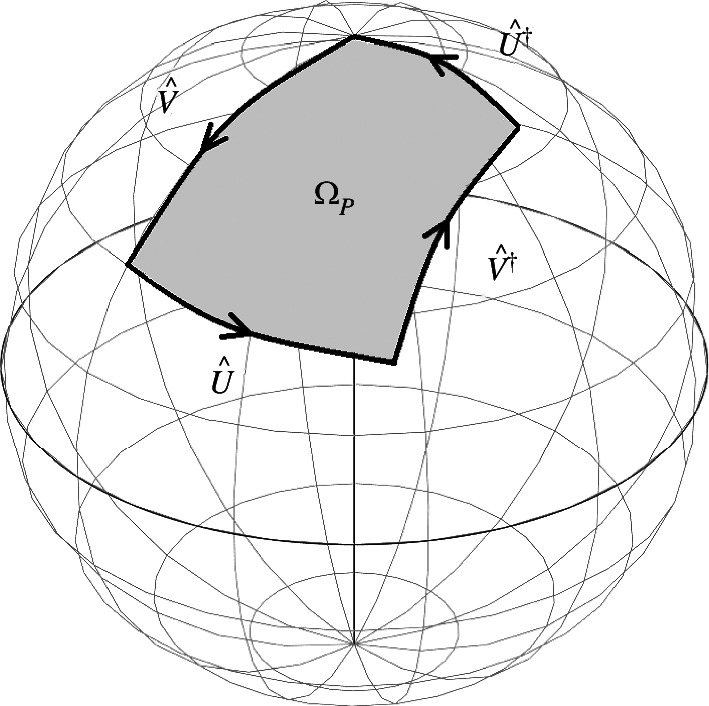
Illustration of the closed path formed by the sequence of transformations ${\hat{U}}^{†}{\hat{V}}^{†}\hat{U}\hat{V}$ on the Bloch or Poincaré sphere. The area enclosed is the Pancharatnam–Berry phase $\Omega_{P}$ associated with the path.

Our task is to demonstrate the advantages to be gained by utilizing indefinite causal order to determine the area on the Bloch sphere enclosed, as illustrated in figure 3 or, equivalently, the Berry phase associated with the sequence of operators ${\hat{U}}^{†}{\hat{V}}^{†}\hat{U}\hat{V}$. At this stage, we seek a general analysis of this rather than one specific to a particular implementation. We consider the example of a device based on an optical Sagnac interferometer in the following sections. To introduce the indefinite causal order we introduce a second qubit which acts as a control qubit initially prepared in the superposition state $\frac{1}{\sqrt{2}}\left(|0\rangle +|1\rangle \right)$. There is then a controlled operation [1,2] such that the two operations are applied in the order $\hat{U}\hat{V}$ if the control qubit is in the state |0⟩ and the order $\hat{V}\hat{U}$ if it is in the state |1⟩:


(2.13) $$\begin{array}{lrlr} & \frac{1}{\sqrt{2}}\left(|0\rangle +|1\rangle \right)|\phi \rangle & \to \frac{1}{\sqrt{2}}\left(|0\rangle ⊗(\hat{U}\hat{V}|\phi \rangle )+|1\rangle ⊗(\hat{V}\hat{U}|\phi \rangle )\right) & \\ & & =\frac{1}{2}\left(|+\rangle ⊗\{\hat{U},\hat{V}\}|\phi \rangle +|-\rangle ⊗\left[\hat{U},\hat{V}\right]|\phi \rangle \right), & \end{array}$$

where $|\pm \rangle =\frac{1}{\sqrt{2}}\left(|0\rangle \pm |1\rangle \right)$, $\left[\hat{U},\hat{V}\right]$ is the commutator $\hat{U}\hat{V}-\hat{V}\hat{U}$, and $\left\{\hat{U},\hat{V}\right\}$ the anticommutator $\hat{U}\hat{V}+\hat{V}\hat{U}$.

If we measure the control qubit in the |±⟩ basis then we find the probabilities


(2.14) P(±)=14⟨ϕ|(U^V^±V^U^)†(U^V^±V^U^)|ϕ⟩=12⟨ϕ|(V^†U^†U^V^±V^†U^†V^U^±U^†V^†U^V^+U^†V^†V^U^)|ϕ⟩=12(1±Re⟨ϕ|U^†V^†U^V^|ϕ⟩)=12(1±cos⁡Ω2),

where we have used equation (2.12).

It is perhaps important to stress that we have used a completely arbitrary input state |ϕ⟩: the result is the same for any input state. Thus, without any information at all about $\hat{U}$ and $\hat{V}$, we can directly measure the Berry phase associated with the closed path enclosed by ${\hat{U}}^{†}{\hat{V}}^{†}\hat{U}\hat{V}$ through the indefinite causal order setup.

We conclude this section with a short proof that the phase Ω is a Berry phase—or more generally, a Pancharatnam phase [33,35,36], when the loop is not closed. The Pancharatnam phase associated with a path


(2.15) $$\begin{array}{lcr} & |A\rangle \to |B\rangle \to |C\rangle \to |D\rangle & \end{array}$$

is given by


(2.16) $$\begin{array}{lcr} & \Omega_{P}=\arg\langle A|B\rangle \langle B|C\rangle \langle C|D\rangle \langle D|A\rangle . & \end{array}$$

In our case, we are interested in the path formed by applying $\hat{V}$, then $\hat{U}$, then ${\hat{V}}^{†}$, and finally ${\hat{U}}^{†}$ as given in (2.11). Thus, we should define the states as follows:


(2.17) $$\begin{array}{rl} |A\rangle & =|\psi \rangle ,\\ |B\rangle & =\hat{V}|\psi \rangle ,\\ |C\rangle & =\hat{U}\hat{V}|\psi \rangle ,\\ |D\rangle & ={\hat{V}}^{†}\hat{U}\hat{V}|\psi \rangle , \end{array}$$

where, as before, we take |ψ⟩ to be the eigenstate of ${\hat{U}}^{†}{\hat{V}}^{†}\hat{U}\hat{V}$ with eigenvalue $e^{i\frac{\Omega }{2}}$. Thus


(2.18) $$\begin{array}{rl} \Omega_{P} & =\arg\langle \psi |\hat{V}|\psi \rangle \langle \psi |{\hat{V}}^{†}\hat{U}\hat{V}|\psi \rangle \langle \psi |{\hat{V}}^{†}{\hat{U}}^{†}{\hat{V}}^{†}\hat{U}\hat{V}|\psi \rangle \langle \psi |{\hat{V}}^{†}{\hat{U}}^{†}\hat{V}|\psi \rangle \\ & =\arg e^{i\frac{\Omega }{2}}\langle \psi |\hat{V}|\psi \rangle \langle \psi |{\hat{V}}^{†}\hat{U}\hat{V}|\psi \rangle \langle \psi |{\hat{V}}^{†}|\psi \rangle \langle \psi |{\hat{V}}^{†}{\hat{U}}^{†}\hat{V}|\psi \rangle \\ & =\arg e^{i\frac{\Omega }{2}}|\langle \psi |\hat{V}|\psi \rangle {|}^{2}|\langle \psi |{\hat{V}}^{†}\hat{U}\hat{V}|\psi \rangle {|}^{2}=\frac{\Omega }{2} \end{array}$$

where, in the second line, we have used the eigenvalue equation for |ψ⟩, (2.11).

The area (or more precisely the solid angle) associated with the closed path generated by the operators $\hat{U}$, $\hat{V}$ and their conjugates is twice the Pancharatnam-Berry phase. Moreover, this phase is simply related to either the commutator or the anticommutator of the unitary operators:


(2.19) $$\begin{array}{rl} \Omega & =2\arccos\left(1-\frac{1}{2}\langle \phi |[{\hat{V}}^{†},{\hat{U}}^{†}][\hat{U},\hat{V}]|\phi \rangle \right)\\ & =2\arccos\left(-1+\frac{1}{2}\langle \phi |\{{\hat{V}}^{†},{\hat{U}}^{†}\}\{\hat{U},\hat{V}\}|\phi \rangle \right). \end{array}$$

The dependence on the commutators or the anticommutators suggests utilizing an optical interferometer to perform the desired measurement as it is known that the output probabilities for single-photon inputs can, with suitable arrangements, depend simply on the commutator or anticommutator of the operations in the interferometer paths [37].

By exploiting indefinite causal order we have seen that it is possible to measure the Pancharatnam–Berry phase with no prior knowledge of the operators $\hat{U}$, and $\hat{V}$, or of the states |ψ⟩ and $|\psi^{⟂}\rangle$, which are the eigenstates of ${\hat{U}}^{†}{\hat{V}}^{†}\hat{U}\hat{V}$.

## 3. Pancharatnam–Berry phase measurement with a Sagnac interferometer

The natural optical implementation of indefinite causal order is the Sagnac interferometer [16], with the unitary transformations realized by waveplates implementing changes of the polarization. This has advantages in terms of stability over other interferometers in that the two optical path lengths (clockwise and anti-clockwise) are naturally balanced. In general, for a waveplate (or sequence of waveplates) implementing the unitary operation in the forward direction, the operation performed when approached in the backward direction is instead


(3.1) $$\begin{array}{lcr} & {\hat{U}}^{\mathrm{bw}}=\hat{P}{\hat{U}}^{T}{\hat{P}}^{†}\neq \hat{U} & \end{array}$$

where $\hat{P}$ is determined by the local choice of coordinates for the forward and backward directions, which may be chosen to be the identity $\hat{P}=\hat{I}$ [16,19]. The transpose is basis dependent and is taken in the |h⟩, |v⟩ basis (horizontal and vertical polarization) once the coordinate system is fixed.

We find it more natural to choose our local coordinates so that the positive z-direction is along the direction of propagation, the y-axis (vertical polarization) is fixed and is orthogonal to the plane of the device, and the x-axis (horizontal polarization) is orthogonal to both, such that together these form a right-handed coordinate system. In this case we find that $\hat{P}=-\hat{Z}$, a polarization implementation of the Pauli Z gate [1,2] in the |h⟩, |v⟩ basis, which acts as follows:


(3.2) $$\begin{array}{rl} \hat{Z}|h\rangle & =|h\rangle \\ \hat{Z}|v\rangle & =-|v\rangle . \end{array}$$

**Figure 4.**
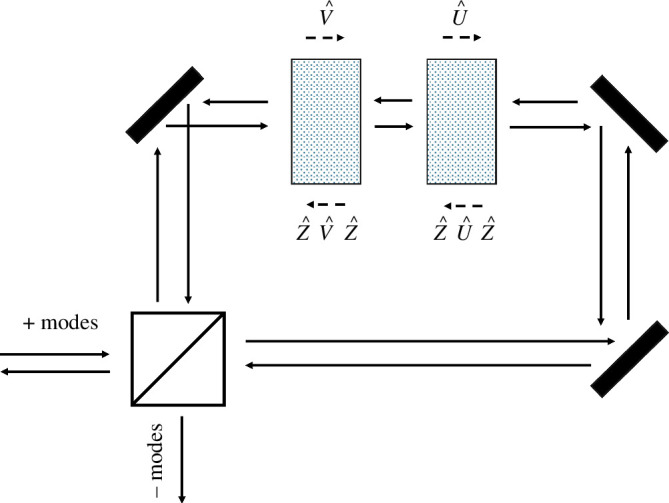
Sagnac interferometer with waveplates implementing transformations $\hat{U}$, $\hat{V}$ when traversed in the clockwise direction, and $\hat{Z}\hat{U}\hat{Z}$, $\hat{Z}\hat{V}\hat{Z}$ in the anti-clockwise direction.

For a single waveplate, ${\hat{U}}_{\mathrm{WP}}^{T}={\hat{U}}_{\mathrm{WP}}$ as may be readily verified by taking the transpose of the Jones matrix for a waveplate [20,38,39]. However, if $\hat{U}$ and $\hat{V}$ are made up of a sequence of waveplates, then their order is reversed under the transpose operation, reflecting the fact that the sequence will be encountered in reverse order, $({\hat{U}}_{1}{\hat{U}}_{2}\ldots {\hat{U}}_{k}{)}^{T}={\hat{U}}_{k}^{T}\ldots {\hat{U}}_{2}^{T}{\hat{U}}_{1}^{T}$. It suffices for our purposes to restrict $\hat{U}$ and $\hat{V}$ to be single waveplates below. Putting these together we therefore find that


(3.3) $$\begin{array}{lcr} & {\hat{U}}^{\mathrm{bw}}=\hat{Z}\hat{U}\hat{Z}, & \end{array}$$

for our setup and choice of coordinates. The setup is illustrated in figure 4.

In our Sagnac interferometer, light propagating in a clockwise direction transforms $\hat{U}\hat{V}$, while light propagating in the anti-clockwise direction experiences the transformation $\hat{Z}\hat{V}\hat{U}\hat{Z}$. It may seem strange that there is an apparent asymmetry between the two paths, with only one depending on $\hat{Z}$, but if we write ${\hat{U}}^{\prime }=\hat{Z}\hat{U}\hat{Z}$ and ${\hat{V}}^{\prime }=\hat{Z}\hat{V}\hat{Z}$, then the anticlockwise path is associated with the transformation ${\hat{V}}^{\prime }{\hat{U}}^{\prime }$ and it is the clockwise transformation that includes the $\hat{Z}$ operators: $\hat{Z}{\hat{U}}^{\prime }{\hat{V}}^{\prime }\hat{Z}$. It follows that in our Sagnac configuration, with waveplates $\hat{U}$, $\hat{V}$, instead of (2.13) we obtain


(3.4) $$\begin{array}{lrlr} & \frac{1}{\sqrt{2}}\left(|0\rangle +|1\rangle \right)|\phi \rangle & \to \frac{1}{\sqrt{2}}\left(|0\rangle ⊗(\hat{U}\hat{V}|\phi \rangle )+|1\rangle ⊗(\hat{Z}\hat{V}\hat{U}\hat{Z}|\phi \rangle )\right) & \\ & & =\frac{1}{2}\left(|+\rangle ⊗\left(\hat{U}\hat{V}+\hat{Z}\hat{V}\hat{U}\hat{Z}\right)|\phi \rangle +|-\rangle ⊗\left(\hat{U}\hat{V}-\hat{Z}\hat{V}\hat{U}\hat{Z}\right)|\phi \rangle \right), & \end{array}$$

where the control qubit from our general analysis corresponds, simply, to the direction of propagation of the light within the interferometer.

To measure the Pancharatnam–Berry phase, recall from (2.14) that we wish to determine the expectation value $\left\langle [\hat{U},\hat{V}{]}^{†}[\hat{U},\hat{V}]\right\rangle$ (or equivalently, the same expression with the commutator replaced with the anti-commutator), and further, that this is *independent of the state* |ϕ⟩ relative to which the expectation value is taken. Thus, we can choose the input state, or input polarization, in any convenient way. It is natural to choose this to be an eigenstate of $\hat{Z}$, corresponding to vertical or horizontal polarization so that the first $\hat{Z}$ has no effect. With the choice |ϕ⟩=|h⟩ we obtain:


(3.5) $$\begin{array}{lcr} & \frac{1}{\sqrt{2}}\left(|0\rangle +|1\rangle \right)|h\rangle \to \frac{1}{2}\left(|+\rangle ⊗\left(\hat{U}\hat{V}+\hat{Z}\hat{V}\hat{U}\right)|h\rangle +|-\rangle \left(\hat{U}\hat{V}-\hat{Z}\hat{V}\hat{U}\right)|h\rangle \right). & \end{array}$$

For a similar reason, we measure the polarization in the output ports on the h/v basis. The probabilities of each combination of output port and polarization are


(3.6) $$\begin{array}{lrlr} & \mathrm{Pr}(+,h) & =\frac{1}{4}|\langle h|\hat{U}\hat{V}+\hat{V}\hat{U}|h\rangle {|}^{2}, & \\ & \mathrm{Pr}(+,v) & =\frac{1}{4}|\langle v|\hat{U}\hat{V}-\hat{V}\hat{U}|h\rangle {|}^{2}, & \\ & \mathrm{Pr}(-,h) & =\frac{1}{4}|\langle h|\hat{U}\hat{V}-\hat{V}\hat{U}|h\rangle {|}^{2}, & \\ & \mathrm{Pr}(-,v) & =\frac{1}{4}|\langle v|\hat{U}\hat{V}+\hat{V}\hat{U}|h\rangle {|}^{2}, & \end{array}$$

where we have used the eigenvalue relation for $\hat{Z}$, (3.2). Finally, we find that the combination Pr(−,h)+Pr(+,v) gives the desired quantity:


(3.7) $$\begin{array}{lrlr} & \mathrm{Pr}(-,h)+\mathrm{Pr}(+,v) & =\frac{1}{4}\left(\langle h|{\left(\hat{U}\hat{V}-\hat{V}\hat{U}\right)}^{†}\left(|h\rangle \langle h|+|v\rangle \langle v|\right)\left(\hat{U}\hat{V}-\hat{V}\hat{U}\right)|h\rangle \right) & \\ & & =\frac{1}{4}\langle h|[\hat{U},\hat{V}{]}^{†}[\hat{U},\hat{V}]|h\rangle & \\ & & =\frac{1}{2}\left(1-\cos\left(\frac{\Omega }{2}\right)\right) & \end{array}$$

where we have used the resolution of the identity |h⟩⟨h|+|v⟩⟨v|=I.

In the above, we assumed the use of a balanced (non-polarizing) beam splitter in the interferometer. In reality, a non-polarizing beamsplitter may also have some non-negligible effect on the polarization degree of freedom, which would cause errors in the desired measurement. An alternative, and experimentally superior, configuration could use a polarizing beam splitter. Choosing the input polarization to be diagonally polarized, to ensure equal contributions in each arm, and assuming that the polarizing beamsplitter transmits horizontal polarization and reflects vertical polarization, we find


(3.8) $$\begin{array}{lrlr} & |0\rangle \frac{1}{\sqrt{2}}\left(|h\rangle +|v\rangle \right) & \to \frac{1}{\sqrt{2}}\left(|0\rangle |v\rangle +|1\rangle |h\rangle \right) & \end{array}$$

where we have labelled the reflected arm |0⟩ and the transmitted arm |1⟩. This setup requires an additional half-wave plate, to ensure that the polarization state on encountering the waveplates is the same in both directions. Thus, we add a half-wave plate in the transmitted arm, to flip |h⟩ to |v⟩. This is equivalent to a Pauli-X operation:


(3.9) $$\begin{array}{rl} \hat{X}|h\rangle & =|v\rangle \\ \hat{X}|v\rangle & =|h\rangle . \end{array}$$

The effect of the whole setup is now


(3.10) $$\begin{array}{lrlr} & \frac{1}{\sqrt{2}}\left(|0\rangle |v\rangle +|1\rangle |h\rangle \right) & \to \frac{1}{\sqrt{2}}\left(|0\rangle ⊗(\hat{X}\hat{U}\hat{V}|v\rangle )+|1\rangle ⊗(\hat{Z}\hat{V}\hat{U}\hat{Z}\hat{X}|h\rangle )\right) & \\ & & =\frac{1}{\sqrt{2}}\left(|0\rangle ⊗(\hat{X}\hat{U}\hat{V}|v\rangle )-|1\rangle ⊗(\hat{Z}\hat{V}\hat{U}|v\rangle )\right). & \end{array}$$

Upon meeting the polarizing beam splitter, once again the horizontal component is transmitted, and the vertical component is reflected. The output ports are labelled so that the |0⟩ path contains the transmitted component of the |0⟩ internal path, and the reflected component of the |1⟩ internal path. Thus


(3.11) $$\begin{array}{lrlr} & \frac{1}{\sqrt{2}}\left(|0\rangle ⊗(\hat{X}\hat{U}\hat{V}|v\rangle )-|1\rangle ⊗(\hat{Z}\hat{V}\hat{U}|v\rangle )\right) & \to \frac{1}{\sqrt{2}}|0\rangle \left(|h\rangle \langle h|\hat{X}\hat{U}\hat{V}|v\rangle -|v\rangle \langle v|\hat{Z}\hat{V}\hat{U}|v\rangle \right) & \\ & & \;+\frac{1}{\sqrt{2}}|1\rangle \left(-|h\rangle \langle h|\hat{Z}\hat{V}\hat{U}|v\rangle +|v\rangle \langle v|\hat{X}\hat{U}\hat{V}|v\rangle \right) & \\ & & =\frac{1}{\sqrt{2}}|0\rangle \left(|h\rangle \langle v|\hat{U}\hat{V}|v\rangle +|v\rangle \langle v|\hat{V}\hat{U}|v\rangle \right) & \\ & & \;+\frac{1}{\sqrt{2}}|1\rangle \left(-|h\rangle \langle h|\hat{V}\hat{U}|v\rangle +|v\rangle \langle h|\hat{U}\hat{V}|v\rangle \right). & \end{array}$$

To obtain the desired interference between the terms corresponding to different causal orders, we need to measure the output on the diagonal (d)/antidiagonal (a) basis. The probabilities of each combination of output port and polarization are now


(3.12) $$\begin{array}{rl} Pr(0,d) & =\frac{1}{4}|\langle v|\hat{U}\hat{V}+\hat{V}\hat{U}|v\rangle {|}^{2},\\ Pr(0,a) & =\frac{1}{4}|\langle v|\hat{U}\hat{V}-\hat{V}\hat{U}|v\rangle {|}^{2},\\ Pr(1,d) & =\frac{1}{4}|\langle h|\hat{U}\hat{V}-\hat{V}\hat{U}|v\rangle {|}^{2},\\ Pr(1,a) & =\frac{1}{4}|\langle h|\hat{U}\hat{V}+\hat{V}\hat{U}|v\rangle {|}^{2}. \end{array}$$

Finally, we find that the combination Pr(0,a)+Pr(1,d) gives the desired quantity:


(3.13) $$\begin{array}{lrlr} & \mathrm{Pr}(0,a)+\mathrm{Pr}(1,d) & =\frac{1}{4}\left(\langle v|{\left(\hat{U}\hat{V}-\hat{V}\hat{U}\right)}^{†}\left(|v\rangle \langle v|+|h\rangle \langle h|\right)\left(\hat{U}\hat{V}-\hat{V}\hat{U}\right)|v\rangle \right) & \\ & & =\frac{1}{2}\left(1-\cos\left(\frac{\Omega }{2}\right)\right), & \end{array}$$

which, once again, gives directly the desired Berry phase.

## 4. Fully quantum theory

In the preceding section, we considered the behaviour of a single photon input into the Sagnac interferometer. We saw that the desired Pancharatnam–Berry phase was revealed in the detection probabilities. It is generally true, however, that such simple single-photon interference experiments give results that are reproduced, precisely, in the classical regime in which a laser is employed [22]. All that is required is to measure the intensities of the various outputs rather than count single photons. It should be noted, however, that other single-photon effects, such as the lack of coincident detections from the diverse outputs, are not reproduced with classical laser fields [40].

If other states of light, such as photon pairs or squeezed light, are to be used then a fully quantum theory is required. We can construct this, simply, by considering the action of the beam splitter and the unitary transformations on the annihilation operators for the various input modes [26]. For brevity, we consider here only the Sagnac interferometer with a non-polarizing beam splitter. The extension to the polarizing version is straightforward.

**Figure 5.**
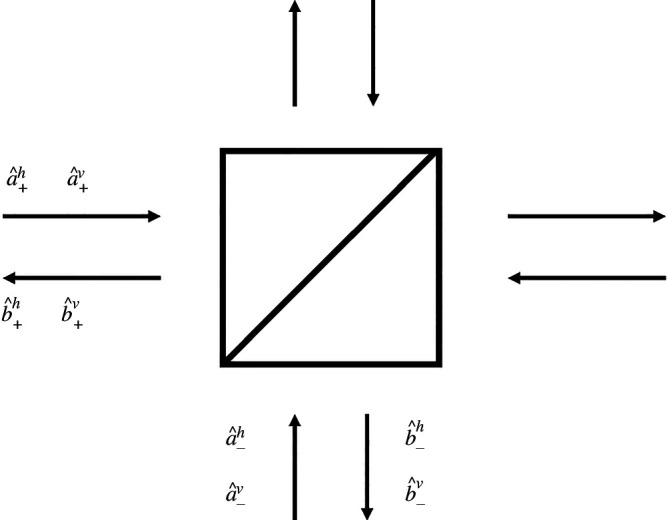
Schematic of a beam splitter used in a Sagnac interferometer, with the arrangement of the input and output annihilation operators of the interferometer labelled, as referred to in the text.


The individual elements, the beam splitter at the input, the sequence of waveplates and the beam splitter again at the output constitute a sequence of unitary transformations on these annihilation operators. In figure 5, we depict the arrangement of the input and output annihilation operators. It is convenient to arrange the four input operators and output operators as column vectors. The relationship between these is then

(4.1) $$\begin{array}{rl} \left(\begin{array}{c} {\hat{b}}_{+}^{h}\\ {\hat{b}}_{+}^{u}\\ {\hat{b}}_{-}^{h}\\ {\hat{b}}_{-}^{u} \end{array}\right) & =\frac{1}{\sqrt{2}}\left(\begin{array}{cccc} 1 & 0 & 1 & 0\\ 0 & 1 & 0 & 1\\ -1 & 0 & 1 & 0\\ 0 & -1 & 0 & 1 \end{array}\right)\left(\begin{array}{cccc} M_{↺}^{hh} & M_{↺}^{hv} & 0 & 0\\ M_{↺}^{vh} & M_{↺}^{vv} & 0 & 0\\ 0 & 0 & M_{↻}^{hh} & M_{↻}^{hv}\\ 0 & 0 & M_{↻}^{vh} & M_{↻}^{vv} \end{array}\right)\frac{1}{\sqrt{2}}\left(\begin{array}{cccc} 1 & 0 & -1 & 0\\ 0 & 1 & 0 & -1\\ 1 & 0 & 1 & 0\\ 0 & 1 & 0 & 1 \end{array}\right)\left(\begin{array}{c} {\hat{a}}_{+}^{h}\\ {\hat{a}}_{+}^{u}\\ {\hat{a}}_{-}^{h}\\ {\hat{a}}_{-}^{u} \end{array}\right) \end{array}$$

where the matrix elements associated with the actions of the waveplates are


(4.2) $$\begin{array}{lrlr} & M_{↺}^{ij} & =\langle i|\hat{Z}\hat{V}\hat{U}\hat{Z}|j\rangle & \\ & M_{↻}^{ij} & =\langle i|\hat{U}\hat{V}|j\rangle . & \end{array}$$

The beam splitter has equal probabilities for transmission and reflection and we have chosen the transmission and reflection coefficients to be real.^2^

As a first illustration of the application of the fully quantum theory, we calculate again the measurement probabilities for a single horizontally polarized photon in the + input. Direct application of (4.1) gives the detection amplitudes:


(4.3) $$\begin{array}{rl} \left(\begin{array}{c} \left\langle 0|{\hat{b}}_{+}^{h}{\hat{a}}_{+}^{h†}|0\right\rangle \\ \left\langle 0|{\hat{b}}_{+}^{v}{\hat{a}}_{+}^{h†}|0\right\rangle \\ \left\langle 0|{\hat{b}}_{-}^{h}{\hat{a}}_{+}^{h†}|0\right\rangle \\ \left\langle 0|{\hat{b}}_{-}^{v}{\hat{a}}_{+}^{h†}|0\right\rangle \end{array}\right) & =\frac{1}{2}\left(\begin{array}{c} M_{↺}^{hh}+M_{↻}^{hh}\\ M_{↺}^{vh}+M_{↻}^{vh}\\ -M_{↺}^{hh}+M_{↻}^{hh}\\ -M_{↺}^{vh}+M_{↻}^{vh} \end{array}\right)=\frac{1}{2}\left(\begin{array}{c} \left\langle h|\hat{U}\hat{V}+\hat{Z}\hat{V}\hat{U}\hat{Z}|h\right\rangle \\ \left\langle v|\hat{U}\hat{V}+\hat{Z}\hat{V}\hat{U}\hat{Z}|h\right\rangle \\ \left\langle h|\left(-\hat{U}\hat{V}+\hat{Z}\hat{V}\hat{U}\hat{Z}\right)|h\right\rangle \\ \left\langle v|\left(-\hat{U}\hat{V}+\hat{Z}\hat{V}\hat{U}\hat{Z}\right)|h\right\rangle \end{array}\right). \end{array}$$

The modulus squared of these gives the probabilities in equation (3.6).

For more complicated input states, it is helpful to have a more general way to analyse the action of our Sagnac interferometer. To that end, we expand the relation (4.1) to write the four output annihilation operators in terms of the four input operators:


(4.4) $$\begin{array}{lrlr} & {\hat{b}}_{+}^{h} & =\frac{1}{2}\left[M_{↺}^{hh}({\hat{a}}_{+}^{h}-{\hat{a}}_{-}^{h})+M_{↻}^{hh}({\hat{a}}_{+}^{h}+{\hat{a}}_{-}^{h})+M_{↺}^{hv}({\hat{a}}_{+}^{v}-{\hat{a}}_{-}^{v})+M_{↻}^{hv}({\hat{a}}_{+}^{v}+{\hat{a}}_{-}^{v})\right] & \\ & {\hat{b}}_{+}^{v} & =\frac{1}{2}\left[M_{↺}^{vh}({\hat{a}}_{+}^{h}-{\hat{a}}_{-}^{h})+M_{↻}^{vh}({\hat{a}}_{+}^{h}+{\hat{a}}_{-}^{h})+M_{↺}^{vv}({\hat{a}}_{+}^{v}-{\hat{a}}_{-}^{v})+M_{↻}^{vv}({\hat{a}}_{+}^{v}+{\hat{a}}_{-}^{v})\right] & \\ & {\hat{b}}_{-}^{h} & =\frac{1}{2}\left[-M_{↺}^{hh}({\hat{a}}_{+}^{h}-{\hat{a}}_{-}^{h})+M_{↻}^{hh}({\hat{a}}_{+}^{h}+{\hat{a}}_{-}^{h})-M_{↺}^{hv}({\hat{a}}_{+}^{v}-{\hat{a}}_{-}^{v})+M_{↻}^{hv}({\hat{a}}_{+}^{v}+{\hat{a}}_{-}^{v})\right] & \\ & {\hat{b}}_{-}^{v} & =\frac{1}{2}\left[-M_{↺}^{vh}({\hat{a}}_{+}^{h}-{\hat{a}}_{-}^{h})+M_{↻}^{vh}({\hat{a}}_{+}^{h}+{\hat{a}}_{-}^{h})-M_{↺}^{vv}({\hat{a}}_{+}^{v}-{\hat{a}}_{-}^{v})+M_{↻}^{vv}({\hat{a}}_{+}^{v}+{\hat{a}}_{-}^{v})\right]. & \end{array}$$

We can use these relationships to determine the full photon counting statistics at the output [26,41]. As an example, we consider two cases in which a pair of photons constitutes the input.

Our first example is the input of two identical photons onto the + port. In this case, the input state can be written in the form:


(4.5) $$\begin{array}{lcr} & |{\mathrm{in}}_{1}\rangle =\frac{1}{\sqrt{2}}{\hat{a}}_{+}^{h†2}|0\rangle . & \end{array}$$

The probabilities that the two photons leave the interferometer in the output ports α and β, with polarizations i and j are


(4.6) $$\begin{array}{lcr} & \mathrm{Pr}(\alpha ,i;\beta ,j)=\frac{1}{1+\delta_{\alpha \beta }\delta_{ij}}\frac{1}{2}{\left|\langle 0|{\hat{b}}_{\alpha }^{i}{\hat{b}}_{\beta }^{j}{\hat{a}}_{+}^{h†2}|0\rangle \right|}^{2}. & \end{array}$$

We can evaluate these using the relationships in equation (4.4). There are 10 distinct probabilities but rather than list these, it suffices to note that in this case, the photons behave, essentially independently so that


(4.7) $$\begin{array}{lcr} & \mathrm{Pr}(\alpha ,i;\beta ,j)=\mathrm{Pr}(\alpha ,i)P(\beta ,j). & \end{array}$$

Our second example is the input of two identical photons, one into the + port and one into the − port. Hence, our input state has the form:


(4.8) $$\begin{array}{lcr} & |{\mathrm{in}}_{2}\rangle =\frac{1}{\sqrt{2}}{\hat{a}}_{+}^{h†}{\hat{a}}_{-}^{h†}|0\rangle . & \end{array}$$

In this case, the photons either both travel clockwise or anticlockwise around the interferometer [26,42–46]. For this reason, we can expect different behaviour from that found for single photon input states because the two input photons both travel the same way around the interferometer. The output probabilities are now


(4.9) $$\begin{array}{lcr} & \mathrm{Pr}(\alpha ,i;\beta ,j)=\frac{1}{1+\delta_{\alpha \beta }\delta_{ij}}{\left|\langle 0|{\hat{b}}_{\alpha }^{i}{\hat{b}}_{\beta }^{j}{\hat{a}}_{+}^{h†}{\hat{a}}_{-}^{h†}|0\rangle \right|}^{2}. & \end{array}$$

It is worth listing the probability amplitudes associated with each of the ten distinct measurement outcomes


(4.10) $$\begin{array}{lrlr} & \frac{1}{\sqrt{2}}\langle 0|{\hat{b}}_{+}^{h2}{\hat{a}}_{+}^{h†}{\hat{a}}_{-}^{h†}|0\rangle & =\frac{1}{2\sqrt{2}}\left(M_{↻}^{hh2}-M_{↺}^{hh2}\right)=\frac{1}{\sqrt{2}}\langle 0|{\hat{b}}_{-}^{h2}{\hat{a}}_{+}^{h†}{\hat{a}}_{-}^{h†}|0\rangle & \\ & \left\langle 0|{\hat{b}}_{+}^{h}{\hat{b}}_{+}^{v}{\hat{a}}_{+}^{h†}{\hat{a}}_{-}^{h†}|0\right\rangle & =\frac{1}{2}\left(M_{↻}^{hh}M_{↻}^{vh}-M_{↺}^{hh}M_{↺}^{vh}\right)=\langle 0|{\hat{b}}_{-}^{h}{\hat{b}}_{-}^{v}{\hat{a}}_{+}^{h†}{\hat{a}}_{-}^{h†}|0\rangle & \\ & \frac{1}{\sqrt{2}}\langle 0|{\hat{b}}_{+}^{v2}{\hat{a}}_{+}^{h†}{\hat{a}}_{-}^{h†}|0\rangle & =\frac{1}{2\sqrt{2}}\left(M_{↻}^{vh2}-M_{↺}^{vh2}\right)=\frac{1}{\sqrt{2}}\langle 0|{\hat{b}}_{-}^{v2}{\hat{a}}_{+}^{h†}{\hat{a}}_{-}^{h†}|0\rangle & \\ & \left\langle 0|{\hat{b}}_{+}^{h}{\hat{b}}_{-}^{h}{\hat{a}}_{+}^{h†}{\hat{a}}_{-}^{h†}|0\right\rangle & =\frac{1}{2}\left(M_{↻}^{hh2}+M_{↺}^{hh2}\right) & \\ & \left\langle 0|{\hat{b}}_{+}^{v}{\hat{b}}_{-}^{v}{\hat{a}}_{+}^{h†}{\hat{a}}_{-}^{h†}|0\right\rangle & =\frac{1}{2}\left(M_{↻}^{vh}M_{↻}^{hh}+M_{↺}^{vh}M_{↺}^{hh}\right) & \\ & \left\langle 0|{\hat{b}}_{+}^{h}{\hat{b}}_{-}^{v}{\hat{a}}_{+}^{h†}{\hat{a}}_{-}^{h†}|0\right\rangle & =\frac{1}{2}\left(M_{↻}^{hh}M_{↻}^{vh}+M_{↺}^{hh}M_{↺}^{vh}\right)=\langle 0|{\hat{b}}_{+}^{v}{\hat{b}}_{-}^{h}{\hat{a}}_{+}^{h†}{\hat{a}}_{-}^{h†}|0\rangle . & \end{array}$$

These amplitudes do not factorize in the same manner as would happen if the two photons entered the interferometer through the same port. It is also apparent that each of the amplitudes is formed from components corresponding to the product of amplitudes with the two photons propagating in the same direction around the interferometer, as is to be expected from the Hong–Ou–Mandel, or the not quite Loudon–Fearn–Rarity–Tapster effect [44]. It is not obvious how these more complicated amplitudes might be related to the single Pancharatnam–Berry phase, but the interference of the *two-photon* amplitudes corresponding to the clockwise and anti-clockwise paths is suggestive of a different topological effect, which may merit further study.

## 5. Conclusion

In this work, we have considered the use of indefinite causal order to measure geometric phases. The use of indefinite order to improve the precision of measurement of an area in phase space was previously proposed in the literature [28], and later experimentally demonstrated [31]. Here we have presented a proposal to measure the solid angle on the Poincaré sphere enclosed by a sequence of unitary transformations of the polarization. We have shown that indefinite causal order allows us to directly measure this solid angle, the Pancharatnam–Berry phase, requiring no prior information about the polarization transformations.

We further discussed a possible optical implementation of this measurement, using a Sagnac interferometer. We have analysed the operation of the device, in a first quantized picture, and finally in a fully quantum second quantized picture. The use of multi-photon interference effects such as the Hong–Ou–Mandel, or the not-quite Loudon–Fearn–Rarity–Tapster effect, is not yet well explored in the field of indefinite causal order. We suggest that further study of the interplay between these two interference effects may lead to novel topological effects and further metrological applications.

## Data Availability

This article has no additional data.

## Footnotes

^1^
It is interesting to note that Sagnac first proposed and realized the interferometer that now bears his name as a challenge to Einstein’s theory of special relativity, in particular the notion of the constancy of the speed of light. It was Langevin who, in 1921, correctly explained the observed effects within the context of general relativity. An account of this controversy may be found in [21].

^2^
Other choices are possible. In particular for a symmetric beam splitter it is usual for the transmission coefficients to be real but for the reflection coefficients to be imaginary.
